# Associations between food consumption with T cell activation and antibody responses following SARS-CoV-2 mRNA vaccination

**DOI:** 10.1080/29933935.2025.2568927

**Published:** 2025-10-25

**Authors:** Hiroki Negishi, Gaku Nakato, Rie Kadowaki, Hiroki Kono, Mami Minakata, Ayumi Ichikawa, Takayuki Toshimitsu, Seiya Makino, Hiroshi Kano, Sho Nakamura, Hiroto Narimatsu, Shinji Fukuda

**Affiliations:** a Wellness Science Labs, Meiji Holdings Co., Ltd, Tokyo, Japan; b Gut Environmental Design Group, Kanagawa Institute of Industrial Science and Technology, Kanagawa, Japan; c Innovative Microbiome Therapy Research Center, Juntendo University Graduate School of Medicine, Tokyo, Japan; d Metagen, Inc, Yamagata, Japan; e R&D Division, Meiji Co., Ltd, Tokyo, Japan; f Graduate School of Health Innovation, Kanagawa University of Human Services, Kanagawa, Japan; g Cancer Prevention and Control Division, Kanagawa Cancer Center Research Institute, Kanagawa, Japan; h Institute for Advanced Biosciences, Keio University, Yamagata, Japan; i Transborder Medical Research Center, Institute of Medicine, University of Tsukuba, Ibaraki, Japan

**Keywords:** COVID-19, fermented foods, gut microbiota, vaccination, yogurt

## Abstract

COVID-19 mRNA vaccines induce protective immunity, but the factors influencing individual immune responses remain incompletely understood. This study investigated associations between dietary habits, T cell activation, and antibody responses to SARS-CoV-2 vaccination. We analyzed peripheral blood mononuclear cells following spike protein stimulation and measured anti-SARS-CoV-2 S protein antibody levels. We found significant positive correlations between CD4^+^ activation-induced marker (AIM)^+^ T cells, CD4^+^ CD69^+^ T cells, and anti-SARS-CoV-2 S protein antibody levels. Among various food items, yogurt and bread consumption frequencies showed significant positive correlations with immune parameters. Participants with high yogurt consumption (daily or more) demonstrated significantly higher levels of CD4^+^ AIM^+^ T cells, CD4^+^ CD69^+^ T cells, and antibody levels compared to low consumption groups. Similar patterns were observed for bread consumption. These associations remained significant after adjusting for age, sex, vaccination count, and prior infection history. Additionally, we observed correlations between serum and fecal anti-SARS-CoV-2 antibodies, with distinct gut microbiota and metabolite profiles associated with antibody levels. Our findings suggest that regular consumption of specific foods may influence vaccine-induced immune responses, potentially through interactions with the gut environment. These results provide a foundation for investigating dietary approaches to optimize vaccine responses.

## Introduction

The development of messenger RNA (mRNA) vaccines has revolutionized the prevention of COVID-19. These vaccines use lipid nanoparticles (LNPs) to deliver modified mRNA encoding the SARS-CoV-2 spike protein, thereby initiating a cascade of innate and adaptive immune responses.^1^^,^^2^ LNPs also function as potent adjuvants, enhancing the immunogenicity of the vaccine platform by activating multiple innate immune pathways.^2^

The immune response to mRNA vaccination involves complex interactions between humoral and cellular immunity. Longitudinal studies have shown that mRNA vaccines induce robust memory B cells and antigen-specific CD4^+^ and CD8^+^ T cells, which exhibit strong proliferative capacity and polyfunctional cytokine production.^3^^,^^4^ Early CD4^+^ T cell responses are particularly crucial, as they orchestrate both humoral and cellular immunity through helper functions.^5^ Although antibody levels naturally decline over time, recent studies have demonstrated that booster doses significantly prolong the half-life of the humoral immune response by 71%−84% compared to the primary vaccination series.^6^ The magnitude and quality of these immune responses vary substantially among individuals and are influenced by factors such as age, pre-existing immunity, and environmental factors.^4^^,^^6^

The gut microbiota has emerged as a crucial modulator of vaccine-induced immune responses. Increasing evidence suggests that gut microbial composition and diversity influence vaccine efficacy through multiple mechanisms, including adjuvant effects and immune regulation.^7–10^ In the context of SARS-CoV-2 vaccination, higher gut microbiota diversity prior to vaccination has been positively correlated with stronger immune responses, particularly in terms of antibody production and T cell activation.^11^

Dietary habits are key determinants of both gut microbiota composition and immune function. Fermented foods, especially yogurt, have been shown to exert immunomodulatory effects.^12^ In a randomized controlled trial, regular consumption of probiotic-containing yogurt enhanced virus-specific IgA production in elderly individuals, suggesting improved mucosal immunity.^13^ Furthermore, a comprehensive dietary intervention study demonstrated that increased consumption of fermented foods enhanced microbiota diversity and reduced inflammatory markers, potentially fostering an environment favorable for robust immune responses.^14^

The relationship between nutrition and immune function is not just limited to fermented foods. Dietary fiber has emerged as a critical modulator of immune responses, primarily through its influence on gut microbiota composition and microbial metabolite production.^15^ Moreover, various micronutrients and food-derived compounds have been shown to affect immune cell function and antibody production.^16^ Recent studies have also highlighted that specific gut bacteria and their metabolites, particularly those influenced by dietary habits, can significantly affect vaccine-induced antibody responses.^11^

Although the importance of diet and the gut microbiota in shaping immune function is well established, the specific mechanisms through which dietary habits modulate vaccine-induced immune responses remain unclear. In particular, the associations between regular consumption of specific foods and the magnitude of vaccine-induced cellular and humoral immunity have yet to be clearly elucidated. Furthermore, the potential of dietary interventions to enhance vaccine efficacy remains largely unexplored.

This study aimed to address these knowledge gaps by examining the associations between dietary habits, particularly the consumption of fermented foods and other dietary components, and SARS-CoV-2 mRNA vaccine-induced immune responses. Specifically, we investigated how patterns of food intake relate to T cell activation and antibody production, and whether these associations may be influenced by the gut microbial environment.

## Methods

### Study design and participants

This cross-sectional study recruited participants from a pool of 349 individuals who had previously undergone antibody testing during the 1-y follow-up survey of the COVID-19 project within the Kanagawa Prospective ‘ME-BYO’ Cohort Study (ME-BYO cohort) in Japan.^17^^,^^18^ Although participants were originally enrolled in the ME-BYO cohort, this substudy was conducted independently. Recruitment materials describing the study were mailed to all individuals who had completed antibody testing in the ME-BYO follow-up survey (as shown in Supplementary Figure 1). Those who were interested registered for participation, and enrollment was limited to the first 111 individuals who successfully completed the registration process. All enrolled participants provided written informed consent at the study site.

Exclusion criteria were applied to minimize potential confounding factors affecting immune responses and to ensure participant safety during blood collection. Individuals were excluded if they: (1) had a history or current diagnosis of autoimmune or immune-related diseases; (2) were pregnant; (3) had contracted any communicable disease within 30 d prior to enrollment; (4) had donated blood components or 200 mL of whole blood within the month preceding study initiation; (5) had donated 400 mL of whole blood within the prior three months (males) or four months (females); (6) would exceed the annual blood donation limit (1200 mL for males, 800 mL for females) if participation in this study were combined with prior donations; or (7) were deemed unsuitable by the principal or co-principal investigators.

### Ethical approval and informed consent

This study was conducted in accordance with the principles outlined in the Declaration of Helsinki and the Ethical Guidelines for Life Science and Medical Research Involving Human Subjects, as issued by the Ministry of Education, Culture, Sports, Science and Technology, the Ministry of Health, Labor and Welfare, and the Ministry of Economy, Trade and Industry of Japan. The study protocol was reviewed and approved by the Ethics Review Committee of Chiyoda Paramedical Care (Institutional Review Board no. 15000088).

All prospective participants received written information detailing the study objectives, methodology, duration, potential risks, and expected benefits. Written informed consent was obtained prior to enrollment. The consent form emphasized that participation was voluntary and that participants could withdraw at any time without consequence. All personal data were anonymized and handled in accordance with institutional privacy policies.

### Sample collection and processing

Peripheral blood mononuclear cells (PBMCs) were isolated from approximately 8 mL of peripheral blood collected into BD Vacutainer tubes containing heparin (BD, USA). Following collection, the tubes were gently inverted eight times to ensure mixing and centrifuged at 1800 × g for 15 min at room temperature within 2 h of collection. The plasma and cell layers were transferred to 15 mL conical tubes using sterile Pasteur pipettes. The cell fraction was further centrifuged at 300 × g for 10 min, washed with PBS, and red blood cells were lysed using ACK buffer for 5 min. After two additional PBS washes, the PBMCs were resuspended in 1 mL of CELLBANKER® 1 plus (ZENOGENPHARM Co., Ltd., Japan), transferred to cryotubes, and stored at −80 °C until further analysis.

For plasma collection, blood was drawn into BD Vacutainer barrier tubes (BD), inverted eight times, and centrifuged at 1800 × g for 15 min at room temperature. Plasma was transferred to 15 mL tubes using and aliquoted in 500 μL volumes in Eppendorf tubes. Samples were then transported to the laboratory under refrigerated conditions and stored at −80 °C until analysis.

Stool samples were collected using an MG kit (Metagen Inc., Japan)^19^ and stored at −80 °C until analysis. Stool samples were available from 27 participants.

### PBMC analysis

PBMCs were seeded at a density of 5 × 10^5^ cells per well and cultured in the presence or absence of 2 µg/mL SARS-CoV-2 (2019-nCoV) Spike S1-His Recombinant Protein (Sino Biological, China) for 24 h under 37 °C in a 5% CO_2_ incubator. Cells were cultured in RPMI 1640 medium (Gibco, USA) supplemented with 5% heat-activated Human Serum AB (GeminiBio, USA). After culturing, cells were collected for flow cytometry analysis and supernatants were collected for measuring IFNγ concentration. IFN-*γ* concentrations in culture supernatants were measured using BD OptEIA™ Human IFN-*γ* ELISA Set (BD) following the manufacturer's protocol without modifications. Samples were initially measured at 1:10 dilution. For samples above the quantification range, 1:100 dilution data were used, and for samples below the quantification range, 1:2 dilution data were used. Values below the detection limit at 1:2 dilution were assigned the lower limit of quantification (9.375 pg/mL).

Cells were stained with the following fluorochrome-conjugated antibodies: CD3-AF700 (clone: UCHT1) (BD), CD4-BV480 (clone: SK3) (BD), CD8-PE-Cy™7 (clone: RPA-T8) (BD), OX40-FITC (clone: ACT35) (Biolegend, USA), CD137- PerCP-Cy™5.5 (clone: 4B4-1) (Biolegend), CD69-BV421 (clone: FN50) (BD), CCR7-PE (clone: 3D12) (BD) and Fixable Viability Dye eFluor™ 780 (Invitrogen, USA). Stained cells were fixed with Cytofix/Cytoperm™ (BD) and acquired using the ID7000™ Spectral Cell Analyzer (Sony, Japan). Data were analyzed using FlowJo software. Flow cytometry gating strategy is detailed in Supplementary Figure 2. Briefly, lymphocytes were identified by scatter characteristics, followed by doublet exclusion and viability discrimination. Activation-induced marker (AIM) – positive cells were defined as OX40^+^ and CD137^+^ populations within CD4^+^ T cells and CD69^+^ and CD137^+^ populations within CD8^+^ T cells, as previously described.^20^

### Antibody measurement

Anti-SARS-CoV-2 antibody levels were measured using the Anti-SARS-CoV-2 S-RBD/Spike protein IgG ELISA Kit (catalog number KE30003, MyBioSource, USA) following the manufacturer's protocol. Plasma samples were diluted 1000-fold using the kit-provided Sample Diluent before analysis. Plasma samples or standards were incubated in precoated wells for 30 min at room temperature, followed by four washes. HRP-conjugated anti-human IgG detection antibody was then added and incubated for an additional 30 min. After another washing step, TMB substrate was applied for 10−15 min to allow color development. The reaction was terminated using stop solution, and absorbance was measured at 450 and 630 nm using a microplate reader. Antibody concentrations were determined based on a standard curve. For fecal samples, 10 mg of sample was suspended in phosphate-buffered saline containing complete EDTA-free protease inhibitor cocktail (Roche, Switzerland) at a final concentration of 2 mg/mL. The supernatant was collected after vortexing and centrifugation at 12,000 rpm for 20 min at 4 °C. Anti-SARS-CoV-2 spike protein-specific IgG and IgA levels in fecal supernatants were measured using the COVID-19 S-Protein (S1RBD) Human IgG ELISA Kit (catalog number ab274340, Abcam) and Human IgA ELISA Kit (catalog number ab276185, Abcam, UK), respectively, following the manufacturer's protocols.

### Questionnaire survey

Background information was collected using comprehensive questionnaires capturing demographic and lifestyle factors. The questionnaire included items on sex, date of birth, age, smoking status, dietary habits, alcohol consumption, exercise patterns, bowel movement frequency, sleep duration, medical history, current medication use, treatment status, allergy history, and COVID-19 vaccination status (including date and vaccine type).

Dietary intake was assessed using a semi-quantitative food frequency questionnaire, which estimated the average food consumption over the preceding six months. Participants reported consumption frequencies using a 10-point scale: 0 = never, 1 = less than once per month, 2 = 1−3 times per month, 3 = 1−2 times per week, 4 = 3−4 times per week, 5 = 5−6 times per week, 6 = once daily, 7 = 2−3 times daily, 8 = 4−6 times daily, and 9 = 7 or more times daily.

### Gut microbiota analysis

Ten milligrams of stool were combined with 0.1 mm zirconia/silica beads in 1% SDS phenol/chloroform/isoamyl alcohol. Samples were disrupted by vigorous shaking at 1500 rpm for 5 min using the Shakemaster (Bio Medical Science Inc., Japan). Bacterial genomic DNA was extracted using a previously described phenol/chloroform/isoamyl alcohol protocol.^21^

The V1–V2 hypervariable regions of the 16S rRNA gene were amplified using a two-step PCR approach. First PCR (20 cycles) used universal bacterial primers with adapter sequences: 27F-mod (5ʹ-ACACTCTTTCCCTACACGACGCTCTTCCGATCT-NNNNN-AGRGTTTGATYMTGGCTCAG−3′) and 338 R (5′-GTGACTGGAGTTCAGACGTGTGCTCTTCCGATCT-NNNNN-TGCTGCCTCCCGTAGGAGT−3′), where NNNNN represents random sequences of 0−5 bases to improve sequencing quality. Second PCR (8 cycles) added indexed primers: 2ndF (5′-AATGATACGGCGACCACCGAGATCTACAC-Index2-ACACTCTTTCCCTACACGACGC−3′) and 2ndR (5′-CAAGCAGAAGACGGCATACGAGAT-Index1-GTGACTGGAGTTCAGACGTGTG−3′). PCR products were purified using VAHTS DNA Clean Beads (Vazyme, China) at 1.5 × reaction volume. Library concentration was measured using QuantiFluor dsDNA System with Synergy H1 (Agilent Technologies, USA), and library quality was assessed using Fragment Analyzer with dsDNA 915 Reagent Kit (Agilent Technologies). Amplicons were sequenced on the MiSeq platform (Illumina, USA) using 2 × 300 bp paired-end sequencing. Sequencing data were processed using Quantitative Insights into Microbial Ecology (QIIME) 2 (version 2023.9.0).^22^ Sequence denoising and trimming were performed using DADA2. Specifically, the first 20 bp of forward reads and 19 bp of reverse reads were removed to eliminate primer sequences. The resulting 135-bp forward and 220-bp reverse reads were used for downstream analysis. Taxonomic classification was performed using the SILVA132 database ^23^^,^^24^ with a Naive Bayesian classifier. For microbiota composition analysis, bacterial taxa with mean relative abundance below 0.25% across all samples were grouped as "Others" to focus on the predominant microbial communities.

### Metabolite analysis

Fecal metabolite extraction and quantification were performed according to previously published protocols.^25^ SCFAs and other organic acids were measured using a 7890 Series gas chromatography-mass spectrometry (GC–MS) system (Agilent Technologies), while bile acids were measured using a 1260 Infinity II liquid chromatography-mass spectrometry (LC–MS) system (Agilent Technologies), following the methods described in a previous study.^26^

### Statistical analysis

All statistical analyzes were conducted using R software (version 4.4.1). Correlations among immune parameters, antibody levels, and food consumption frequencies were assessed using Spearman's rank correlation coefficient to account for non-normally distributed data. Statistical significance was defined as *p *< 0.05.

To determine optimal thresholds for food consumption frequency, receiver operating characteristic (ROC) curve analysis was conducted. For each immune parameter – CD4^+^ AIM^+^ T cells, CD4^+^ CD69^+^ T cells, and anti-SARS-CoV-2 S protein antibody – sensitivity, specificity, and Youden's index (defined as sensitivity + specificity − 1) were calculated across all possible cutoff values. The cutoff value that maximized Youden's index was selected as the optimal threshold to categorize participants into high- and low-consumption groups.

Differences in immune parameters between high and low food consumption groups were compared using the Wilcoxon rank-sum test.

To assess independent associations between food consumption and immune parameters, multivariate linear regression models were constructed, adjusting for age, sex, vaccination count, and SARS-CoV-2 infection history within the past year.

Hierarchical clustering was performed using Ward's method to identify groups of food items with similar correlation patterns in relation to immune responses. All graphs were generated using the ggplot2 package in R.

### Results

### Participant characteristics and study overview

We first examined the anti-SARS-CoV-2 S protein antibody levels in a cohort of 349 individuals who received COVID-19 vaccination (Supplementary Figure 1). The cohort included individuals with varying vaccination histories (0–5 doses) and time intervals post-vaccination. Antibody levels exhibited substantial inter-individual variability, which remained evident even after adjusting for the number of vaccine doses received.

From this larger cohort, we recruited 111 participants to explore factors associated with vaccine-induced immune responses. Participants had received varying numbers of COVID-19 vaccine doses (range: 0−5 doses), primarily consisting of mRNA vaccines (Pfizer-BioNTech and Moderna). Sample collection occurred at various time points post-vaccination (range: 22−571 d).

### T cell responses and antibody correlations

**Figure 1.**
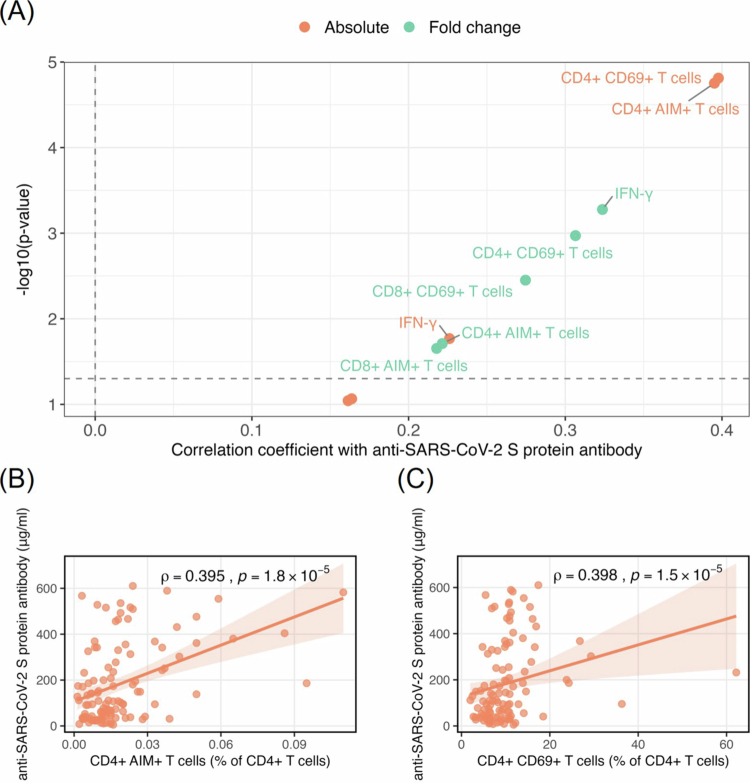
Association between SARS-CoV-2 spike protein-specific T cell responses and anti-SARS-CoV-2 S protein antibody levels. (A) Volcano plot showing the correlation between spike protein-specific T cell responses following SARS-CoV-2 spike protein stimulation of PBMCs and anti-SARS-CoV-2 S protein antibody levels. The x-axis shows Spearman's correlation coefficients, and the y-axis shows statistical significance as −log10 (*p*-value). Orange dots represent absolute values after stimulation, and blue dots represent fold changes from baseline. The horizontal dashed line indicates *p* = 0.05. (B, C) Scatter plots showing the correlation between spike protein-induced CD4 + AIM + T cells (B) or CD4 + CD69 + T cells (C) and anti-SARS-CoV-2 S protein antibody levels. Lines indicate linear regression with 95% confidence intervals (shaded area). Spearman's correlation coefficients (*ρ*) and *p*-values are shown in the upper right corner of each plot.

Analysis of immune parameters in PBMCs stimulated with SARS-CoV-2 spike protein identified several variables significantly positively correlated with anti-SARS-CoV-2 S protein antibody levels (Figure 1). These included both absolute values and fold changes from baseline in CD4^+^ AIM^+^ T cells, CD4^+^ CD69^+^ T cells, CD8^+^ AIM^+^ T cells, and IFN-*γ* production. IFN-*γ* levels were measured in culture supernatants from unstimulated and spike protein-stimulated PBMCs. Unstimulated samples showed median IFN-*γ* levels of 9.38 pg/mL (range: 9.375−21743.8 pg/mL), while spike-stimulated samples showed elevated median levels of 546.26 pg/mL (range: 9.375−30701.4 pg/mL). The median fold change in IFN-*γ* production following stimulation was 11.88 (range: 0.28−2107.93). Among these, CD4^+^ AIM^+^ (*ρ* = 0.395, *p* = 1.8 × 10^−5^) and CD4^+^ CD69^+^ (*ρ* = 0.398, *p* = 1.5 × 10^−5^) T cells exhibited the strongest positive correlations with antibody titers.

### Dietary associations with immune parameters

**Figure 2.**
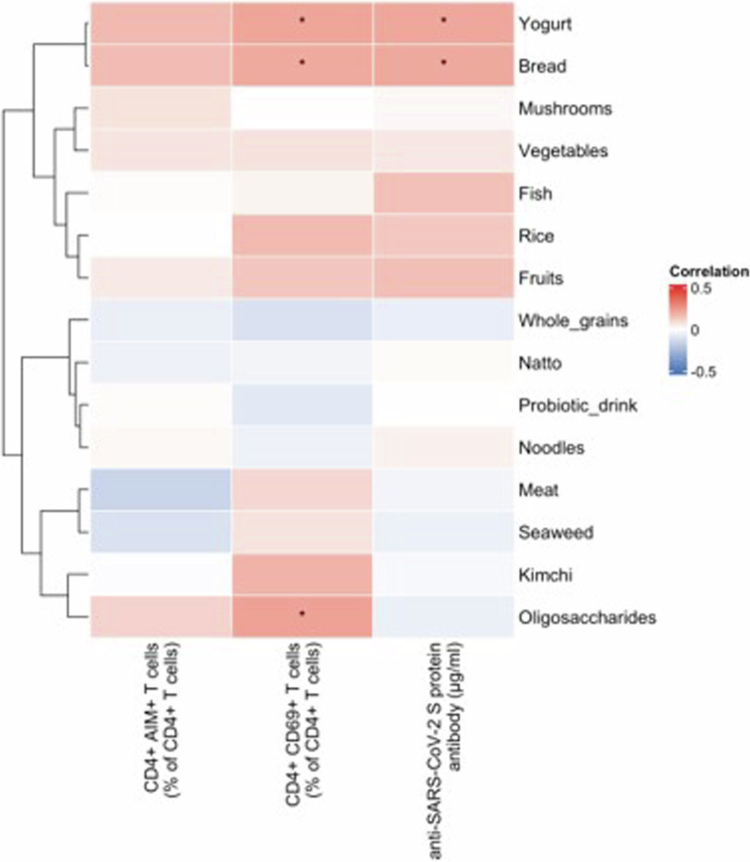
Correlation analysis of food consumption frequency with SARS-CoV-2 spike protein-specific T cell responses and antibody levels. Heatmap showing Spearman's correlation coefficients between food consumption frequency and immune responses to SARS-CoV-2. T cell responses were measured after SARS-CoV-2 spike protein stimulation of PBMCs, and anti-SARS-CoV-2 S protein antibody levels were measured in plasma. The dendrogram on the left shows hierarchical clustering of food items based on their correlation patterns (Ward's method). The color scale indicates correlation strength, ranging from negative (blue) to positive (red). Asterisks indicate statistical significance: *p < 0.05.

To investigate the potential influence of diet on vaccine responses, we evaluated associations between food consumption frequency and immune parameters (Figure 2). Heatmap analysis of 15 food items revealed distinct correlation patterns. Hierarchical clustering identified groups of foods with similar association profiles. Among fermented foods, yogurt showed significant positive correlations with CD4^+^ AIM^+^ T cells, CD4^+^ CD69^+^ T cells, and anti-SARS-CoV-2 S protein antibody levels. Although natto and probiotic beverages clustered with fermented foods, their associations with immune parameters were not statistically significant. Bread consumption also significantly and positively associated with CD4^+^ CD69^+^ T cells and antibody levels. Other fermented foods, including kimchi, demonstrated variable but non-significant correlations. The remaining food categories, such as vegetables, fish, and meat, showed weak or no significant associations.

### Food consumption group comparisons

Receiver operating characteristic (ROC) curve analyzes were performed to determine optimal consumption thresholds for distinguishing high versus low dietary exposure groups (Supplementary Figure 3). For yogurt, optimal cut-off values were 5.5 for CD4^+^ AIM^+^ and CD4^+^ CD69^+^ T cells (corresponding to daily consumption or more), and 4.5 for antibody levels (5−6 times per week or more). For bread, the optimal thresholds were 4.5 for CD4^+^ AIM^+^ and CD4^+^ CD69^+^ T cells (5−6 times per week or more) and 5.5 for antibody levels (daily consumption or more). Consumption frequency distributions are shown in Supplementary Figure 4.

**Figure 3.**
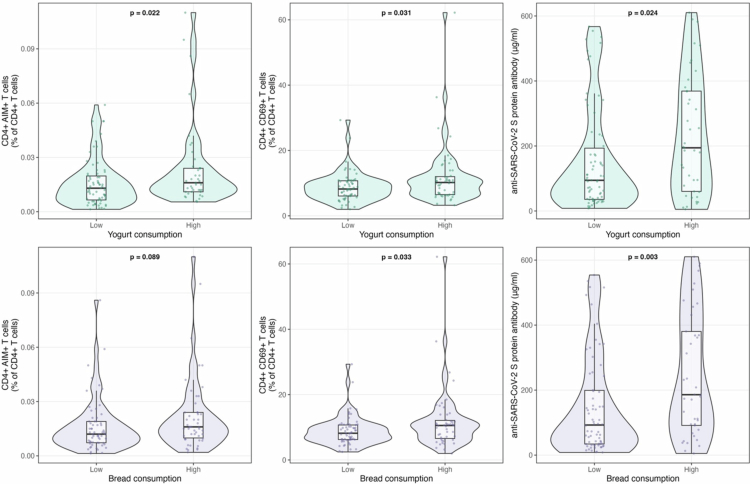
Group comparisons of immune parameters by food consumption. Box plots showing the differences in immune parameters between high and low consumption groups for yogurt and bread. Groups were divided based on optimal cutoff values determined by ROC analysis (Supplementary Figure 3). The box shows the interquartile range (IQR) with the median line; whiskers extend to 1.5 × IQR. Individual data points are shown as jittered dots. Statistical significance was determined using the Wilcoxon rank-sum test. CD4 + T cell subsets are expressed as percentages of total CD4 + T cells, and anti-SARS-CoV-2 S protein antibody levels are shown in μg/mL.

When participants were stratified into high- and low-consumption groups based on ROC-derived cutoff values, distinct patterns in immune parameters emerged (Figure 3). Participants with higher yogurt consumption showed significantly increased levels of CD4^+^ AIM^+^ T cells (*p* = 0.022), CD4^+^ CD69^+^ T cells (*p* = 0.031), and anti-SARS-CoV-2 S protein antibodies (*p* = 0.024) compared to those in the low-consumption group. For bread consumption, significant differences between the high and low consumption groups were observed specifically in CD4^+^ CD69^+^ T cells (*p* = 0.033) and anti-SARS-CoV-2 S protein antibody levels (*p* = 0.003), whereas CD4^+^ AIM^+^ T cell levels were not significantly different between the groups.

### Multivariate analysis

**Figure 4.**
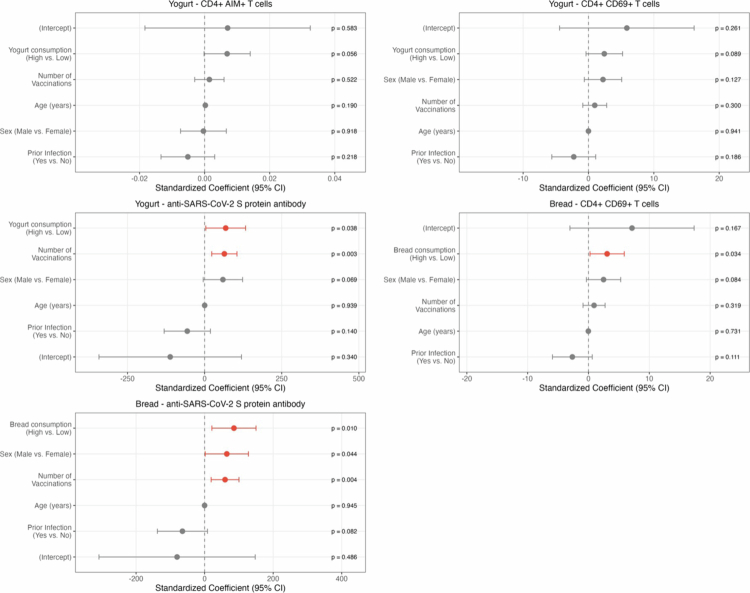
Multivariate analysis results for each immune parameter. Forest plots showing standardized coefficients with 95% confidence intervals from multiple linear regression analyzes. The effects of yogurt and bread consumption on each immune response parameter are presented separately. The models were adjusted for age, sex, vaccination count, and history of infection. Red points and bars indicate statistically significant associations (*p *< 0.05). *p*-values are shown for each variable in the model.

Multivariate analyzes, adjusted for age, sex, vaccination count, and prior SARS-CoV-2 infection within the past year, confirmed these associations (Figure 4). In yogurt consumption analysis, the association with anti-SARS-CoV-2 S protein antibody levels remained significant after adjustment. Similarly, bread consumption was independently associated with elevated CD4^+^ CD69^+^ T cells and antibody levels.

### Gut microbiota and metabolite associations

**Figure 5.**
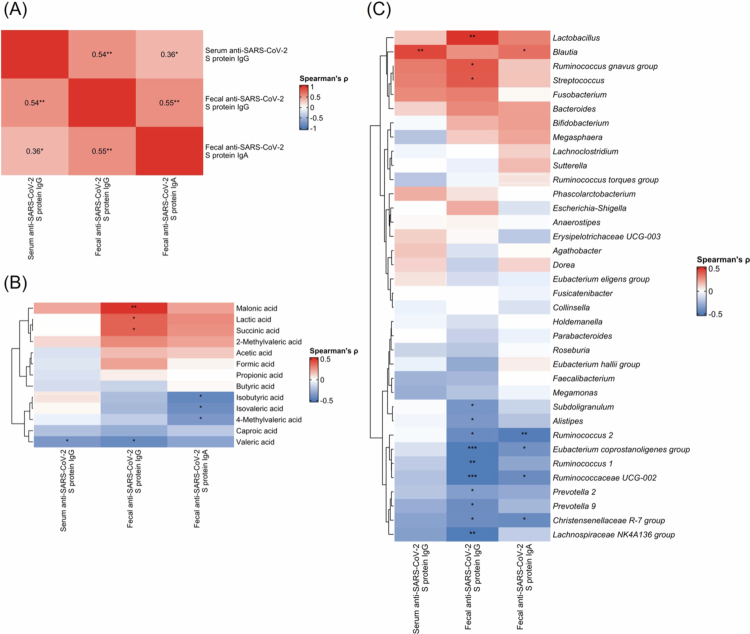
Correlation analysis of serum and fecal antibodies with gut microbiota and metabolites. (A) Correlation matrix showing relationships between serum anti-SARS-CoV-2 S protein IgG, fecal anti-SARS-CoV-2 S protein IgG, and fecal anti-SARS-CoV-2 S protein IgA levels. (B) Heatmap displaying correlations between gut microbiota composition and antibody levels. (C) Heatmap showing correlations between fecal metabolites and antibody levels. Color scales represent Spearman's correlation coefficients (*ρ*). Asterisks indicate statistical significance: *p < 0.05, **p < 0.01, ***p < 0.001. Hierarchical clustering was performed using Ward's method.

Analysis of the relationship between antibody responses and gut environment revealed multiple significant correlations. The correlation analysis demonstrated positive relationships between serum anti-SARS-CoV-2 S protein IgG, fecal anti-SARS-CoV-2 S protein IgG, and fecal anti-SARS-CoV-2 S protein IgA levels, with correlation coefficients ranging from 0.36 to 0.55 (Figure 5A).

Metabolomic profiling revealed that malonic acid, lactic acid, and succinic acid levels were significantly and positively correlated with fecal anti-SARS-CoV-2 S IgG (Figure 5B). In contrast, valeric acid levels were negatively associated with both serum and fecal IgG. Fecal IgA levels showed significant negative correlations with isobutyric acid, isovaleric acid, and 4-methylvaleric acid.

Gut microbiota analysis identified several bacterial taxa associated with antibody levels (Figure 5C). *Blautia* demonstrated a significant positive correlation with serum and fecal IgG (Figure 5C). In addition, *Lactobacillus*, *Ruminococcus gnavus*, and *Streptococcus* were positively associated with fecal IgG levels. Conversely, *Ruminococcus* 2, *Eubacterium coprostanoligenes*, Ruminococcaceae UCG-002, Christensenellaceae R-7, and Lachnospiraceae NK4A136 were negatively correlated with fecal IgG and IgA levels. Individual participant data for fecal metabolites, antibody levels, and microbiota composition are presented in Supplementary Table.

## Discussion

In this study, we identified three key findings regarding immune responses to SARS-CoV-2 vaccination. First, we found strong correlations between T cell activation markers and anti-SARS-CoV-2 spike protein IgG antibody levels, with CD4^+^ AIM^+^ and CD69^+^ T cells showing particularly strong associations. Second, higher frequencies of yogurt and bread consumption were positively associated with both cellular and humoral immune responses. Third, we observed significant correlations between serum and fecal anti-SARS-CoV-2 antibody levels, suggesting a link between systemic and mucosal immunity.

The relationship between T cell responses and antibody production aligns with the current understanding of immune coordination. AIM assays performed following spike protein stimulation of PBMCs have proven to be reliable for detecting antigen-specific T cell responses and offer valuable insights into vaccine-induced immunity.^27^ Moreover, CD69 expression is essential for the development and maintenance of memory T helper cells, which play an essential role in sustaining long-term humoral immunity.^28^

The observed associations between yogurt consumption and immune parameters are consistent with previous research demonstrating that fermented foods influence gut microbiota stability and composition.^29^ These foods exhibit anti-inflammatory and immunomodulatory effects through mechanisms involving lactic acid-producing bacteria and bioactive compounds.^30^ Our findings suggest that regular intake of fermented dairy products may enhance vaccine-induced immune responses via gut-immune interactions.

Regular consumption of bread with diverse dietary fibers has been shown to modify gut microbiota composition and metabolic profiles.^31^ Furthermore, the baking process itself may affect the immunological properties of bread. Comparative research has demonstrated that baked bread can modify cytokine expression and immune-related metabolic pathways compared to steamed bread.^32^ In addition to fiber, wheat flour components such as gluten and gliadin have been shown to activate CD4 T cells.^33^^,^^34^ However, as none of our participants had celiac disease (individuals with autoimmune diseases were excluded), these gluten-related immune effects may not be directly applicable to our healthy population. Additionally, our dietary questionnaire assessed general bread consumption without distinguishing between different types of bread (e.g., white bread, whole grain bread). Therefore, we cannot definitively characterize the fiber content of the bread consumed by participants. Future studies should include more detailed dietary assessments to distinguish between different bread types and their specific nutritional compositions. Overall, these findings suggest that both the composition and processing of bread may contribute to its immunomodulatory effects.

The correlation between serum and fecal anti-SARS-CoV-2 antibody levels observed in our study highlights the intricate relationship between systemic and mucosal immunity. As SARS-CoV-2 primarily infects the upper respiratory tract through the nasal passages, mucosal immune responses, particularly the production of secretory IgA, play a crucial role in early viral defense.^35^ These findings also suggest that fecal antibody measurements could serve as a noninvasive method for monitoring vaccine-induced immune responses, with potential utility in large-scale immunological studies and longitudinal surveillance.

Our analysis of fecal metabolites and microbiota composition further supports the relationship between the gut environment and vaccine-induced immunity. The positive correlation between succinic acid and antibody levels aligns with recent findings indicating that succinic responds to immunization and is linked to enhanced antibody production.^36^ Similarly, the association between lactic acid and antibody levels may reflect the immunomodulatory effects of lactic acid-producing bacteria, which are known to support mucosal immune function.^37^ Notably, the positive correlation between *Blautia* abundance and antibody levels is consistent with emerging evidence identifying *Blautia* as a microbial marker of enhanced vaccine responsiveness.^38^^,^^39^ The consistent associations observed between specific bacterial taxa and antibody levels in our study support the hypothesis that gut microbiota composition may influence host immune responses to vaccination.^10^

This study has several limitations that should be considered when interpreting the results. First, as an observational study, we cannot infer causality between dietary habits and vaccine responses. Second, food consumption was assessed using self-reported frequency questionnaires, which are subjected to recall bias and inaccuracies in portion size estimation.^40^^,^^41^ Importantly, our study evaluated consumption frequency but did not assess portion sizes or absolute quantities consumed, which could significantly influence the observed associations between dietary habits and immune responses. Third, although we adjusted for several major confounding factors – including age, sex, vaccination count, and prior infection history – other lifestyle variables that may affect immune responses, such as sleep quality, stress, and broader dietary patterns, were not assessed.^42^ Given the complex interactions between lifestyle factors and immune function described in previous research,^31^ future studies should adopt more comprehensive and integrative designs.

Future research should address several key questions raised by our findings. Prospective interventional studies are needed to evaluate whether dietary modifications, particularly increased consumption of fermented foods and fiber-rich products, can enhance vaccine-induced immune responses. Mechanistic investigations are also warranted to elucidate how diet-induced shifts in gut microbiota composition and metabolism modulate immune function. In addition, the correlation between serum and fecal antibody levels highlights the need for further research into the role of mucosal immunity in systemic vaccine protection.

In conclusion, this study identified associations between dietary habits and vaccine-induced immune responses, with a particular focus on fermented food and bread consumption. These findings suggest that dietary factors may influence immune responses to vaccination through multiple mechanisms, including modulation of T cell activation and coordination between systemic and mucosal compartments. While further studies are necessary to establish causality and clarify the underlying biological pathways, our results lay the groundwork for investigating dietary approaches to optimize vaccine efficacy.^43^^,^^44^

## Supplementary Material

Supplementary Material Supplementary Table. Individual fecal metabolite, antibody and microbiota data

## Data Availability

The sequenced microbiome 16S rRNA data have been deposited in the DDBJ Sequence Read Archive Repository (accession number PRJDB20505).

## References

[1] Lee Y, Jeong M, Park J, Jung H, Lee H. Immunogenicity of lipid nanoparticles and its impact on the efficacy of mRNA vaccines and therapeutics. Exp Mol Med. 2023;55:2085–2096. doi: 10.1038/s12276-023-01086-x. 37779140 PMC10618257

[2] Verbeke R, Hogan MJ, Loré K, Pardi N. Innate immune mechanisms of mRNA vaccines. Immunity. 2022;55:1993–2005. doi: 10.1016/j.immuni.2022.10.014. 36351374 PMC9641982

[3] Goel RR, Painter MM, Apostolidis SA, Mathew D, Meng W, Rosenfeld AM, Lundgreen KA, Reynaldi A, Khoury DS, Pattekar A, et al. mRNA vaccines induce durable immune memory to SARS-CoV-2 and variants of concern. Science. 2021;374:1979. doi: 10.1126/science.abm0829. PMC9284784 34648302

[4] Gao F, Mallajosyula V, Arunachalam PS, van der Ploeg K, Manohar M, Röltgen K, Yang F, Wirz O, Hoh R, Haraguchi E, et al. Spheromers reveal robust T cell responses to the Pfizer/BioNTech vaccine and attenuated peripheral CD8+ T cell responses post SARS-CoV-2 infection. Immunity. 2023;56:864–878.e4. doi: 10.1016/j.immuni.2023.03.005. 36996809 PMC10017386

[5] Painter MM, Mathew D, Goel RR, Apostolidis SA, Pattekar A, Kuthuru O, Baxter AE, Herati RS, Oldridge DA, Gouma S, et al. Rapid induction of antigen-specific CD4+ T cells is associated with coordinated humoral and cellular immunity to SARS-CoV-2 mRNA vaccination. Immunity. 2021;54:2133–2142.e3. doi: 10.1016/j.immuni.2021.08.001. 34453880 PMC8361141

[6] Korosec CS, Dick DW, Moyles IR, Watmough J. SARS-CoV-2 booster vaccine dose significantly extends humoral immune response half-life beyond the primary series. Sci Rep. 2024;14:8426. doi: 10.1038/s41598-024-58811-3. 38637521 PMC11026522

[7] Hong SH. Influence of microbiota on vaccine effectiveness: “is the microbiota the key to vaccine-induced responses? J Microbiol. 2023;61:483–494. doi: 10.1007/s12275-023-00044-6. 37052795 PMC10098251

[8] Hirota M, Tamai M, Yukawa S, Taira N, Matthews MM, Toma T, Seto Y, Yoshida M, Toguchi S, Miyagi M, et al. Human immune and gut microbial parameters associated with inter-individual variations in COVID-19 mRNA vaccine-induced immunity. Commun Biol. 2023;6:368. doi: 10.1038/s42003-023-04755-9. 37081096 PMC10119155

[9] Lunken GR, Golding L, Schick A, Majdoubi A, Lavoie PM, Vallance BA. Gut microbiome and dietary fibre intake strongly associate with IgG function and maturation following SARS-CoV-2 mRNA vaccination. Gut. 2022;73:208–210. doi: 10.1136/gutjnl-2022-328556. PMC10715502 36549875

[10] Feng Y, de Jong SE, Oliveira APBN, Samaha H, Yang F, Hu M, Wang Y, Beydoun N, Xie X, Zhang H, et al. Antibiotic-induced gut microbiome perturbation alters the immune responses to the rabies vaccine. Cell Host Microbe. 2025;33:705–718. e5. doi: 10.1016/j.chom.2025.03.015. 40252648 PMC12084132

[11] Liu L, He X, Wang J, Li M, Wei X, Yang J, Cheng G, Du W, Liu Z, Xiao X. Exploring the associations between gut microbiota composition and SARS-CoV-2 inactivated vaccine response in mice with type 2 diabetes mellitus. mSphere. 2024;9(9), e0038024. doi: 10.1128/msphere.00380-24. 39189780 PMC11423585

[12] Meydani SN, Ha W-K. Immunologic effects of yogurt. Am J Clin Nutr. 2000;71:861–872. doi: 10.1093/ajcn/71.4.861. 10731490

[13] Yamamoto Y, Saruta J, Takahashi T, To M, Shimizu T, Hayashi T, Morozumi T, Kubota N, Kamata Y, Makino S, et al. Effect of ingesting yogurt fermented with Lactobacillus delbrueckii ssp. bulgaricus OLL1073R-1 on influenza virus-bound salivary IgA in elderly residents of nursing homes: a randomized controlled trial. Acta Odontol Scand. 2019;77:517–524. doi: 10.1080/00016357.2019.1609697. 31094267

[14] Wastyk HC, Fragiadakis GK, Perelman D, Dahan D, Merrill BD, Yu FB, Topf M, Gonzalez CG, Van Treuren W, Han S, et al. Gut-microbiota-targeted diets modulate human immune status. Cell. 2021;184:4137–4153.e14. doi: 10.1016/j.cell.2021.06.019. 34256014 PMC9020749

[15] Cohen Y, Elinav E. Dietary fibers & immunity—more than meets the eye. Cell Res. 2023;33:411–412. doi: 10.1038/s41422-022-00770-3. 36646763 PMC10235049

[16] Munteanu C, Schwartz B. The relationship between nutrition and the immune system. Front Nutr. 2022;9: 1082500. doi: 10.3389/fnut.2022.1082500. 36570149 PMC9772031

[17] Sawaguchi E, Nakamura S, Watanabe K, Tsuno K, Ikegami H, Shinmura N, Saito Y, Narimatsu H. COVID-19-related stigma and its relationship with mental wellbeing: a cross-sectional analysis of a cohort study in Japan. Front Public Health. 2022;10: 1010720. doi: 10.3389/fpubh.2022.1010720. 36249227 PMC9558281

[18] Nakamura S, Watanabe R, Saito Y, Watanabe K, Chung U Il, Narimatsu H. The ME-BYO index: A development and validation project of a novel comprehensive health index. Front Public Health. 2023;11: 1142281. doi: 10.3389/fpubh.2023.1142281. 37213649 PMC10196396

[19] Nomaguchi T, Yamauchi Y, Nishimoto Y, Togashi Y, Ito M, Salim F, Fujisawa K, Murakami S, Yamada T, Fukuda S. Optimized sampling method for fecal microbiome and metabolome preservation under room temperature. medRxiv. 2023. doi: 10.1101/2023.05.08.23289643.

[20] Dan JM, Mateus J, Kato Y, Hastie KM, Yu ED, Faliti CE, Grifoni A, Ramirez SI, Haupt S, Frazier A, et al. Immunological memory to SARS-CoV-2 assessed for up to 8 months after infection. Science. 2021;371:1979. doi: 10.1126/science.abf4063. PMC7919858 33408181

[21] Murakami S, Goto Y, Ito K, Hayasaka S, Kurihara S, Soga T, Tomita M, Fukuda S. The consumption of bicarbonate-rich mineral water improves glycemic control. Evid Based Complement Altern Med. 2015;2015:1–10. doi: 10.1155/2015/824395. PMC4698932 26798400

[22] Bolyen E, Rideout JR, Dillon MR, Bokulich NA, Abnet CC, Al-Ghalith GA, Alexander H, Alm EJ, Arumugam M, Asnicar F, et al. Reproducible, interactive, scalable and extensible microbiome data science using QIIME 2. Nat Biotechnol. 2019;37:852–857. doi: 10.1038/s41587-019-0209-9. 31341288 PMC7015180

[23] Yilmaz P, Parfrey LW, Yarza P, Gerken J, Pruesse E, Quast C, Schweer T, Peplies J, Ludwig W, Glöckner FO. The SILVA and ‘all-species Living Tree Project (LTP)’ taxonomic frameworks. Nucleic Acids Res. 2014;42:D643–D648. doi: 10.1093/nar/gkt1209. 24293649 PMC3965112

[24] Quast C, Pruesse E, Yilmaz P, Gerken J, Schweer T, Yarza P, Peplies J, Glöckner FO. The SILVA ribosomal RNA gene database project: Improved data processing and web-based tools. Nucleic Acids Res. 2013;41:D590–D596. doi: 10.1093/nar/gks1219. 23193283 PMC3531112

[25] Yang J, Song I, Saito M, Hartanto T, Ichinohe T, Fukuda S. Partially hydrolyzed guar gum attenuates symptoms and modulates the gut microbiota in a model of SARS-CoV-2 infection. Gut Microbiome. 2025;6 e1. doi: 10.1017/gmb.2024.7. 39944118 PMC11810603

[26] Hashimoto S, Tochio T, Funasaka K, Funahashi K, Hartanto T, Togashi Y, Saito M, Nishimoto Y, Yoshinori M, Nakaoka K, et al. Changes in intestinal bacteria and imbalances of metabolites induced in the intestines of pancreatic ductal adenocarcinoma patients in a Japanese population: a preliminary result. Scand J Gastroenterol. 2023;58:193–198. doi: 10.1080/00365521.2022.2114812. 36036243

[27] Poloni C, Schonhofer C, Ivison S, Levings MK, Steiner TS, Cook L. T-cell activation–induced marker assays in health and disease. Immunol Cell Biol. 2023;101:491–503. doi: 10.1111/imcb.12636. 36825901 PMC10952637

[28] Shinoda K, Tokoyoda K, Hanazawa A, Hayashizaki K, Zehentmeier S, Hosokawa H, Iwamura C, Koseki H, Tumes DJ, Radbruch A, et al. Type II membrane protein CD69 regulates the formation of resting T-helper memory. Proc Natl Acad Sci U S A. 2012;109:7409–7414. doi: 10.1073/pnas.1118539109. 22474373 PMC3358871

[29] Jeyaram K, Lahti L, Tims S, Heilig HGHJ, van Gelder AH, de Vos WM, Smidt H, Zoetendal EG. Fermented foods affect the seasonal stability of gut bacteria in an Indian rural population. Nat Commun. 2025;16:771. doi: 10.1038/s41467-025-56014-6. 39824829 PMC11748640

[30] Shahbazi R, Sharifzad F, Bagheri R, Alsadi N, Yasavoli-Sharahi H, Matar C. Anti-inflammatory and immunomodulatory properties of fermented plant foods. Nutrients. 2021;13:1516. doi: 10.3390/nu13051516. 33946303 PMC8147091

[31] Ranaivo H, Thirion F, Béra-Maillet C, Guilly S, Simon C, Sothier M, Van Den Berghe L, Feugier-Favier N, Lambert-Porcheron S, Dussous I, et al. Increasing the diversity of dietary fibers in a daily-consumed bread modifies gut microbiota and metabolic profile in subjects at cardiometabolic risk. Gut Microbes. 2022;14. doi: 10.1080/19490976.2022.2044722. PMC8942430 35311446

[32] Wang H, Pang G. Baked bread enhances the immune response and the catabolism in the human body in comparison with steamed bread. Nutrients. 2020;12:1. doi: 10.3390/nu12010001. PMC7019488 31861252

[33] Rahmani S, Galipeau HJ, Clarizio AV, Wang X, Hann A, Rueda GH, Kirtikar UN, Constante M, Wulczynski M, Su H-M, et al. Gluten-dependent activation of CD4+ T cells by MHC Class II–expressing epithelium. Gastroenterology. 2024;167:1113–1128. doi: 10.1053/j.gastro.2024.07.008. 39128638

[34] Mazzarella G. Effector and suppressor T cells in celiac disease. World J Gastroenterol. 2015;21:7349. doi: 10.3748/wjg.v21.i24.7349. 26139981 PMC4481430

[35] Russell MW, Mestecky J. Mucosal immunity: The missing link in comprehending SARS-CoV-2 infection and transmission. Front Immunol. 2022;13: 957107. doi: 10.3389/fimmu.2022.957107. 36059541 PMC9428579

[36] He M, Huang Y, Wang Y, Liu J, Han M, Xiao Y, Zhang N, Gui H, Qiu H, Cao L, et al. Metabolomics-based investigation of SARS-CoV-2 vaccination (Sinovac) reveals an immune-dependent metabolite biomarker. Front Immunol. 2022;13. doi: 10.3389/fimmu.2022.954801. PMC9554639 36248825

[37] Vilander AC, Dean GA. Adjuvant strategies for lactic acid bacterial mucosal vaccines. Vaccines. 2019;7:150. doi: 10.3390/vaccines7040150. 31623188 PMC6963626

[38] Seong H, Choi BK, Han Y, Kim JH, Gim JA, Lim S, Noh JY, Cheong HJ, Kim WJ, Song JY. Gut microbiota as a potential key to modulating humoral immunogenicity of new platform COVID-19 vaccines. Signal Transduct Target Ther. 2023;8:178. doi: 10.1038/s41392-023-01445-0. 37137906 PMC10154741

[39] Ray S, Narayanan A, Vesterbacka J, Blennow O, Chen P, Gao Y, Gabarrini G, Ljunggren HG, Buggert M, Manoharan L, et al. Impact of the gut microbiome on immunological responses to COVID-19 vaccination in healthy controls and people living with HIV. NPJ Biofilms Microbiomes. 2023;9:104. doi: 10.1038/s41522-023-00461-w. 38123600 PMC10733305

[40] Shim J-S, Oh K, Kim HC. Dietary assessment methods in epidemiologic studies. Epidemiol Health. 2014;36:e2014009. doi: 10.4178/epih/e2014009. 25078382 PMC4154347

[41] Bailey RL. Overview of dietary assessment methods for measuring intakes of foods, beverages, and dietary supplements in research studies. Curr Opin Biotechnol. 2021;70:91–96. doi: 10.1016/j.copbio.2021.02.007. 33714006 PMC8338737

[42] Zimmermann P, Curtis N. Factors that influence the immune response to vaccination. Clin Microbiol Rev. 2019;32. doi: 10.1128/CMR.00084-18. PMC6431125 30867162

[43] Calder PC. Nutrition and immunity: lessons for COVID-19. Eur J Clin Nutr. 2021;75:1309–1318. doi: 10.1038/s41430-021-00949-8. 34163017 PMC8220366

[44] Rayman MP, Calder PC. Optimising COVID-19 vaccine efficacy by ensuring nutritional adequacy. Br J Nutr. 2021;126:1919–1920. doi: 10.1017/S0007114521000386. 33504378 PMC7884658

